# With Our Editors

**Published:** 1906-06-15

**Authors:** 


### WITH OUR EDITORS.

**CRITICISMS OF DENTAL COLLEGES.** From time to time we read of aged spinsters with cork-screw curls and childless married ladies, adepts in the practice of race suicide, meeting in solemn convention and laying down rules for the guidance of what they are pleased to call ignorant mothers regarding the proper way to rear children. Akin to this are the criticisms we frequently hear of dental colleges from men untrained as educationalists, and without any experience in the teaching or management of such institutions.

The colleges, as at present conducted, do not in any way meet with their approval, but they are particularly severe regarding their business methods, their entrance requirements and the way in which they graduate students. If a new laboratory be established, they claim it is for the fee received and not for the benefit of the student. If the course of study be lengthened, it is considered a mercenary act and not one with a view to a higher standard of education. Everything in which a fee is involved is criticized, as if an institution, however honorable the work in which it may be engaged, can live without income.

While it is desirable that the entrance requirements to every profession should be high, there is a limit at which they become exclusive. Those without responsibility may urge such standards as being ideal, but the practical common sense of those in authority know that it is visionary to advance beyond what existing conditions will warrant.

The graduation of students is always a serious problem to college faculties, notwithstanding the ease with which self-imposed critics dispose of this question. To the superficially bright and showy student his classmate of slower growth often appears dull and unworthy of graduation, but those who have experience in teaching and examining know well that while the former type of student too often reaches his full stature on graduation day, many a one who has "blossomed late" continues to grow during his whole professional career.

It seems strange that among the many critics of dental colleges a sufficient number do not organize and establish a college after their own ideals—one in which the business element is entirely eliminated, the entrance requirements raised to an exclusive standard, and a fixed percentage of the graduating class plucked regardless of their standing. Until they show that the maintenance of such a college is practicable, we shall continue to regard their criticisms with the same amusement as we do the work of the ladies mentioned in the opening paragraph.—*Odontoblast.*

**DENTAL MEDICINES.** It is gratifying to note the rising wave of protest against those dental preparations with Pe-ru-na-like names that have been placed on the market within the past few years. Possessing no therapeutic value beyond that of some well-known drug they contain, their merits are laudet in such well-phrased language that ignorant dentists are found in numbers who are willing to pay exorbitant prices for them believing that theyare specific in certain dental diseases.

Closely allied to this class, but without the offensive names are the local anesthetics offered for sale. Depending on cocain for their anesthetic properties, with, perhaps, an antiseptic and a heart stimulent added, they are guaranteed to possess such superior qualities, that, besides making operations absolutely painless, no bad after-effects ever result. Here again the cost is a hundred-fold the value of the simple drugs used. When cocain tablets of definite amounts can be obtained from responsible manufacturers, it is certainly more satisfactory to prepare solutions of required strength immediately before the anesthetic is used.

A third objectionable class is that, apparently like the reputable proprietary medicine, with the name of the drugs entering into the preparation printed on each bottle. They differ, however, in this regard that some of the drugs mentioned are not to be found in the Pharmacopœia. Enquiry of the manufacturers will elicit the reply that "they are drugs compounded by their special chemist after years of exhaustive study." As a rule, these firms have associated with them as stock holders "leading" dentists throughout the country who, sharing in the dividends, are expected to recommend the preparations to their patients.

It is not astounding that a portion of the public should be sufficiently credulous to be a prey of nostrum vendors, but it seems incredible that they should gain the confidence of any member of a profession requiring years of study in materia medica.—*Odontoblast.*

**RELIEF FOR SAN. FRANCISCO.** The more we get of the details of the situation at San Francisco the worse it seems. At least 400 dentists have had their entire equipment destroyed, and their source of revenue cut off. In many respects it will be worse than building up a new practice. Their patients are scattered and many of them gone for good. It will take months at the shortest for any of them to become self-sustaining. Let the profession all over the land stop and think what this means and then act accordingly. Fortunately it is only necessary for dentists to understand the situation to make them rise to the occasion and do their duty, and there seems to have been a spontaneous response from the profession everywhere. The Chicago committee has already raised in this vicinity about $4,000. But the work must not stop till every San Francisco man is placed firmly on his feet. To do anything short of this under existing circumstances is a reflection on the manhood of the profession and we should see to it that there is no necessity for any of our brothers to accept assistance from any source except from dentists. It should be a family matter with us and should appeal to our pride in a purely professional sense. The San Francisco men are too brave and high spirited to let their real wants be prominently known, and it rests with us to take the initiative and generously offer our aid.—*Review.*

**HIGH FEES.** This is not a plee for low fees. It is a protest against those Philistines who would measure professional knowledge and skill by the fees received. The matter of fees is almost entirely a business affair. The dentist with well-developed commercial instinct is sure to secure high fees, however inefficient his service may be, while one of superior attainments often fails to receive adequate remuneration through lack of business tact.

High or exorbitant fees can be obtained only from the rich. Now, the wealthy class, as a rule, are not the people of the highest intelligence, and it is doubtful if a dentist, however much time he may devote to study and experiment in order to perfect himself in his profession, would attract their attention by such a course. To gain a wealthy clientele he must camp on their trail and follow them to their social haunts. He must frequent the fashionable church, or rather churches, for a dentist after patronage must be qualified to pray with equal fervor in the church of Jew or gentile. He must become a member of the leading clubs and "mix" in genial fellowship incidentally making known the location of his office. He must be found on the golf links, ostensibly for health, but in reality to keep in touch with the money-bags. He must be exclusive in receiving patients, for fear that Mrs. Society might lose caste did she sit in the same dental chair as some self-reliant working-girl.

But to secure such a class of patients with attending high fees costs money, takes up much valuable time, and unfits one for the performance of intelligent, high-class service. Moreover, the net income from such a practice may often make a poor showing when compared with one in which more modest fees have been charged.

The really great men in dentistry are not associated with the fees they received. Miller's name will ever be honored for his discovery of the cause of dental caries; Black's for his many scientific investigations; Taft's for his high standing as a dental educationalist. How much more satisfactory is this than to enjoy a moment's brief notoriety, as a dentist that pulled Battenberg's leg for a thousand dollars.—*Odontoblast.*

**COLLEGES AND STATE BOARDS.** In the May issue of the *Review* we published a brief history of the Wisconsin case, with extracts from the Supreme Court decisions, in which the powers and limitations of the Board of Examiners in that State are very clearly drawn so far as their jurisdiction over college is concerned. It was practically a service of notice on the board that its duty lay in securing information as to the fitness of a candidate to practice dentistry rather than to supervise the curriculum and establish rules for the conduct of colleges. This decision places the colleges and the board in a more enlightened relationship to each other, and we trust it may result in a better understanding of the rights and duties of all concerned.

It seems to us that the time is now ripe for a most careful consideration of this entire question, to the end that the prolonged contention between the boards and colleges may be satisfactorily adjusted. This contention has given rise to much bitterness, has cost thousands of dollars for litigation, and has really stood in the way of the best advancement of dental education.

That some colleges have been inefficient no one will question, but that others have been struggling hard at the greatest possible expenditure of energy on the part of their faculties to bring their instruction to the highest degree of perfection is equally true. That some boards have been manifestly unfair and even dishonest has been amply proved, and others have been composed of men of the highest honor and ability. But this does not justify the attitude on the part of the board that all colleges are open to suspicion, and should have the rod held over them, any more than it does that the colleges should consider all boards antagonistic.

What is needed at this time is a frank coming together of all parties concerned, and a free discussion as to the rights and privileges of the boards and colleges, with a determination on the part of all to so harmonize matters that dental educational institutions shall not be hampered as they are and have been. C. N. Johnson, in *Review.*

**DENTAL TACTICS.** Although a great many books have been written to guide the medical practitioner on the road to success in practice, the latest, of which is the work of a German doctor, such is not the case so far as our own specialty is concerned.

In making this statement, we are not losing sight of the fact that a great many useful articles and papers have, from time to time, appeared, in which this subject has been dealt with. The matter is an important one, for it has been well observed that success in medical practice requires other qualifications, in addition to a knowledge of medical science. That, in point of fact, medicine is an art. This statement is naturally true to an even greater degree of dental surgery, but in either profession a knowledge of mankind and of the world is of incalculable value. The accuracy of such an assertion is, it is to be feared, recognized by many recently qualified practitioners who on leaving their hospitals, immediately commence practice for themselves. They very soon discover that there is a vast difference between this and dealing with hospital patients.

One of the many advantages of the old system of apprenticeship, which has absolutely died out, so far as medical pupilage is concerned, was that at the very threshold of his career, so to speak, the student was brought in contact with patients and saw how his principal's experience had taught him to deal with their little idiosyncracies. For a similar reason it is to be regretted that the system of dental hospital apprenticeship appears to be supplanting, to a considerable extent, the older plan of the student being articled to a registered dental practitioner. When this newer plan has been allowed to take the place of the old, and the young diplomat, in his hurry to make a way for himself, omits to spend a few years as operating assistant to an older practitioner, the requisite knowledge of the world and its ways has to be acquired in the expensive and hard school of experience.

For these reasons it may be profitable to some among our readers, more especially perhaps the younger members if we refer, somewhat briefly though it must be, to some points in this publication which seem to commend themselves as likely to act as guiding beacons in the troubled waters of dental practice. Foremost, then, is the point, too often overlooked, especially after a few years, that first impressions tell; and probably in no path of life to a greater degree than dental practice. For this reason Dr. Schlesinger advises punctilious personal cleanliness and orderliness in dress without any tendency to elegance, and counsels the avoidance of any eccentricity of manner.

This may strike the casual observer as quite superfluous advice, but only the other day we heard of a West End London dentist who performs his extractions clad in a smock, and previous to the operation informed his patient that he was a perfect butcher when he once began to extract.

Again, he is warned to be firm and decided in his statements and his directions. There is no good, for instance, in telling a patient that he must lose a tooth or have it crowned instead of filled, as he desired, or start the wearing of a denture as though the dentist himself were the person most in need of advice on the subject.

He is also enjoined to remember that his calling is the most exacting of all professions, making large demands upon his health and strength, and that, therefore, he should avoid excesses of all kinds, since he may at any moment be brought face to face with difficult situations requiring all his faculties at their best; he should likewise refrain from gambling, speculation or other outside interests involving stress or anxiety, but should seek diversion in artistic, dramatic or musical pursuits, or in sporting or manly exercises.

Dr. Schlesinger propounds the aphoristic piece of advice that to be a patient listener is a matter of the greatest importance, and warns against the attempt to impress by making a rapid diagnosis, strongly recommending a thorough and detailed examination in all cases.

In our own specialty, though naturally not in the same degree, the adjustment of the treatment to the particular needs of an individual patient requires all the knowledge of human nature that a man can acquire. The restless, the anxious and the heedless types of persons need widely different models of procedure if the best possible result is to be obtained in each case.

The particular temperament and disposition will often have to chiefly determine the practitioner in his decision as to whether to attempt the conservation of a tooth or pulp or at once condemn it.

It is recommended that the directions given should always be clear and free from ambiguity or any possiblity of misconception, and should be repeated and written down if necessary.

Were this rule followed by the dentist, we should not hear so often of patients sleeping every night without removing their dentures and of others applying hot fomentation to their faces.

Secret preparations should be discountenanced, but the use of domestic remedies is not necessarily to be banned.

In regard to professional secrecy, he is most decided that to this there shall be no exceptions, and recommends the most extreme circumspection not only in reference to information given to other practitioners, but to relatives of the patient.

It is to be feared that a great many dentists are much in need of such an admonition—*i. e.*, that they are given to gossiping to their patients concerning not only the *personal* of their *clientele* but actually about what they do for them. It is no excuse for them to say that they never mentioned the fact that any particular individual is the wearer of artificial teeth, a point on which some people are so unduly sensitive. Once a dentist begins to talk about what he does for this or that patient, and is prepared to answer questions put to him on such a subject, the refusal or unwillingness to discuss any particular case at once betrays the reason for this.—*Dental Surgeon.*

